# White paper by the Society for CSF Analysis and Clinical Neurochemistry: Overcoming barriers in biomarker development and clinical translation

**DOI:** 10.1186/s13195-018-0359-x

**Published:** 2018-03-15

**Authors:** Charlotte E. Teunissen, Markus Otto, Sebastiaan Engelborghs, Sanna-Kaisa Herukka, Sylvain Lehmann, Piotr Lewczuk, Alberto Lleó, Armand Perret-Liaudet, Hayrettin Tumani, Martin R. Turner, Marcel M. Verbeek, Jens Wiltfang, Henrik Zetterberg, Lucilla Parnetti, Kaj Blennow

**Affiliations:** 1 Neurochemistry Lab and Biobank, Department of Clinical Chemistry, Amsterdam Neuroscience, VU University Medical Center Amsterdam, Amsterdam, The Netherlands; 2 Department of Neurology, University of Ulm, Ulm, Germany; 3 Reference Center for Biological Markers of Dementia (BIODEM), University of Antwerp, Antwerp, Belgium; 4 Department of Neurology and Memory Clinic, Hospital Network Antwerp (ZNA) Middelheim and Hoge Beuken, Antwerp, Belgium; 5 Department of Neurology, University of Eastern Finland and Kuopio University Hospital, Kuopio, Finland; 6 Université de Montpellier, University Hospital, INSERM U1183, Montpellier, France; 7 Department of Psychiatry and Psychotherapy, Universitätsklinikum Erlangen, Erlangen, Germany; 8 Friedrich-Alexander Universität Erlangen-Nürnberg, Erlangen, Germany; 9 Department of Neurodegeneration Diagnostics, Medical University of Białystok, Białystok, Poland; 10 Department of Biochemical Diagnostics, University Hospital of Białystok, Białystok, Poland; 11 Memory Unit, Department of Neurology, Institut d’Investigacions Biomèdiques Sant Pau, Hospital de Sant Pau, Universitat Autònoma de Barcelona, Barcelona, Spain; 12 Centro de Investigación Biomédica en Red en Enfermedades Neurodegenerativas, CIBERNED, Madrid, Spain; 13 Neurobiology Laboratory, Department of Biochemistry and Molecular Biology, Hospices Civils de Lyon, Lyon, France; 14 University of Lyon 1, CNRS UMR5292, INSERM U1028, BioRan, Lyon, France; 15 Department of Neurology, CSF Laboratory, MS Outpatient Unit, University Hospital of Ulm, Ulm, Germany; 16 Specialty Hospital of Neurology Dietenbronn, Acadamic Hospital of University of Ulm, Schwendi, Germany; 17 Nuffield Department of Clinical Neurosciences, University of Oxford, Oxford, UK; 18 Radboud University Medical Center, Donders Institute for Brain, Cognition and Behaviour, Departments of Neurology and Laboratory Medicine, Radboud Alzheimer Centre, Nijmegen, The Netherlands; 19 Department of Psychiatry and Psychotherapy, University Medical Center (UMG), Georg-August University, Goettingen, Germany; 20 German Center for Neurodegenerative Diseases (DZNE), Goettingen, Germany; 21 iBiMED, Medical Sciences Department, University of Aveiro, Aveiro, Portugal; 22 Clinical Neurochemistry Laboratory, Sahlgrenska University Hospital, Mölndal, Sweden; 23 Department of Psychiatry and Neurochemistry, Institute of Neuroscience and Physiology, the Sahlgrenska Academy at the University of Gothenburg, Mölndal, Sweden; 24 Department of Molecular Neuroscience, UCL Institute of Neurology, Queen Square, London, UK; 25 UK Dementia Research Institute at UCL, London, UK; 26 Center for Memory Disturbances, Lab of Clinical Neurochemistry, Section of Neurology, Department of Medicine, University of Perugia, Perugia, Italy

**Keywords:** Society, Education Biomarkers, Neurology, Cerebrospinal fluid, Body fluids, Biomarker discovery, Assay development, Clinical implementation

## Abstract

Body fluid biomarkers have great potential for different clinical purposes, including diagnosis, prognosis, patient stratification and treatment effect monitoring. This is exemplified by current use of several excellent biomarkers, such as the Alzheimer’s disease cerebrospinal fluid (CSF) biomarkers, anti-neuromyelitis optica antibodies and blood neurofilament light. We still, however, have a strong need for additional biomarkers and several gaps in their development and implementation should be filled. Examples of such gaps are i) limited knowledge of the causes of neurological diseases, and thus hypotheses about the best biomarkers to detect subclinical stages of these diseases; ii) the limited success translating discoveries obtained by e.g. initial mass spectrometry proteomic low-throughput studies into immunoassays for widespread clinical implementation; iii) lack of interaction among all stakeholders to optimise and adapt study designs throughout the biomarker development process to medical needs, which may change during the long period needed for biomarker development.

The Society for CSF Analysis and Clinical Neurochemistry (established in 2015) has been founded as a concerted follow-up of large standardisation projects, including BIOMARKAPD and SOPHIA, and the BioMS-consortium.

The main aims of the CSF society are to exchange high level international scientific experience, to facilitate the incorporation of CSF diagnostics into clinical practice and to give advice on inclusion of CSF analysis into clinical guidelines. The society has a broad scope, as its vision is that the gaps in development and implementation of biomarkers are shared among almost all neurological diseases and thus they can benefit from the activities of the society.

## Background

### Introduction to the use of biomarkers for neurological diseases

Biomarkers in body fluids, such as cerebrospinal fluid (CSF) and blood, play an important role in clinical practise of neurological diseases. For example, CSF analysis of 14-3-3 proteins and aggregated prion proteins provides the final diagnosis for sporadic Creutzfeldt Jakob’s disease [1], and the presence of oligoclonal IgG bands has been the cornerstone of multiple sclerosis diagnosis [2]. Likewise, the discovery of antibodies against aquaporin 4 has completely changed the diagnostic criteria of neuromyelitis optica (NMO) and significantly enhanced knowledge on pathogenesis [3]. More recently, amyloid Aβ42, tTau and pTau levels are positioned at the core of the diagnostic guidelines for Alzheimer’s disease (AD). Appropriate use criteria (AUC) for these AD CSF biomarkers are under development, which is a project led by the Alzheimer’s Association. Moreover, biomarkers are increasingly important due to evidence that there is a long presymptomatic pathological phase in neurological diseases. This had been recognised for a long time for Parkinson’s disease, for which we know that motor features become detectable only after a 60% reduction in striatal neurons [4]. In addition, the CSF biomarkers amyloid Aβ42, tTau and pTau, more and more used in clinical practise, appear to decline up to 25 years before clinical symptoms are visible [5, 6]. The Aβ42/Aβ40 ratio is useful for differentiating AD from Dementia with Lewy Bodies (DLB) patients independent of the technology [7, 8] or increases the concordance with amyloid measurement, in comparison to Aβ42 alone [9]. In addition, the increase in pTau is quite specific for AD compared to other dementia types [10]. These biomarkers very consistently show marked differences in sporadic AD dementia and moreover in prodromal AD [11]. This has led to the prominent use of these biomarkers in guidelines for defining preclinical diagnosis of AD and to increase the confidence in the diagnosis in the prodromal and dementia phases [12, 13]. Even though early treatment may be absent, currently for several neurological diseases, a biomarker-based diagnosis can help to better inform patients and caregivers, and will be important in treatment development [14].

CSF biomarkers, as they are in close interaction with brain tissue, can give a glimpse into brain biochemistry and the underlying pathophysiology of brain disorders such as AD. Biomarkers measured in such body fluids should therefore help us to track pathological events at early stages. Likewise, an important current concept is that the full diversity of mechanisms occurring in pre-clinical stages of neurological diseases are reflected in the CSF’s molecular make up, and possibly also in blood. These biomarkers can therefore be used to support early diagnosis and prognosis, as implemented for the biomarkers amyloid Aβ42, tTau and pTau for AD [12, 13]. Another important use of such biomarkers is in stratification of patients in clinical trials, where patients with the targeted pathology can be selectively treated [15–18]. For example, patients that are amyloid positive (low CSF amyloid Aβ42 values) will be the ones that will benefit from amyloid targeting drugs such as BACE inhibitors, anti-tau immunisation, etc. [16]. On top of that, biomarkers will play an even more important role in secondary prevention trials, i.e. trials aimed at prevention of pathological progression in asymptomatic subjects with positive biofluid or imaging biomarkers. So far, all epidemiological studies and the few intervention studies for dementia have been based on clinically diagnosed dementia. Therefore, epidemiological studies employing biomarkers will be key to identify risk factors for neurodegeneration. Further, a biomarker-based diagnosis will be the cornerstone to identify patients with preclinical disease during the long prodromal stage of neurological diseases. Moreover, no clinical outcome measures will be available in this preclinical phase by definition, when patients are selected based on biomarker profiles. Fluid biomarkers can then be used to monitor treatment effects on pathologically relevant pathways, such as changes in Aβ production and in Aβ species in BACE inhibitor trials [19, 20], or reduced neurofilament light chain levels in aggressive anti-inflammatory treatment in multiple sclerosis [21–23].

Blood-based central nervous system-specific biomarkers are also upcoming and the expectation is that they will aid in prescreening at-risk subjects. Due to the lower invasiveness and absence of a risk for headache of a venepuncture, blood-based biomarkers are strongly preferred for population screening and monitoring requiring repeated sampling. With advances in novel ultrasensitive technologies, analysis of brain-specific proteins in the blood compartment becomes within reach. The high expectation that blood-based biomarker analysis for neurodegenerative and neuroinflammatory diseases will provide information on brain pathology in individual patients is fuelled by recent findings on blood levels of the axonal dysfunction biomarker neurofilament light. The blood levels of this protein correlate strongly with CSF levels [24–26], are increased in a broad range of neurodegenerative diseases [24–31]) and are almost as sensitive as CSF analysis to monitor therapeutic responses in individual patients [21, 32].

## Need for biomarkers for neurological disorders

Evidence for the clinical benefit of AD CSF biomarkers suggests that even more specific CSF and blood biomarkers for neurological disorders, to address the unmet clinical need for early differential diagnosis and disease monitoring, have yet to be discovered. However, not many novel biomarkers identified in past years have reached clinical implementation, to meet all medical needs. Neurofilaments in the differential diagnosis of ALS [33] or the presence of anti-aquaporin 4 antibodies in neuromyelitis optica [34, 35] are quite sensitive and specific, but only when applied in the correct diagnostic context. For example, neurofilaments are non-disease-specific biomarkers, with increases seen in Amyotrophic Lateral Sclerosis (ALS) when compared to slowly progressive disorders [33]. Thus, the comparison or control group defines the clinical value. Markers that are specific for a diagnosis even outside a narrow differential diagnostic spectrum, like the detection of pathological prion protein with the RT-QuIC assay [36], are still not within reach.

We need to identify the factors that could be underlying this development gap and we can learn from positive examples from the past. Important lessons can be learnt from the path by which the AD biomarkers have been developed; discovered in the 1990s, they are now, almost 25 years later, facing implementation in routine clinical chemistry platforms.

As agreed in working groups during the past two decades, one of the causes of the lack of novel clinically useful biomarkers was thought to be the lack of standardisation of biobanking protocols and assay validation, hampering sufficiently powered biomarker discovery studies and replication of initial findings. However, these drawbacks were addressed by for example developing standardised protocols and enhanced knowledge of the effects of preanalytical factors and variation in biobanking protocols on body fluid biomarkers [37–39] and by developing guidelines for assay development and validation [40]. The AD biomarker studies have determined the importance of tube types—it is crucial to use polypropylene vials [41]—and that these should be filled to enhance the volume to surface ratio [42]. Adaptation of the collection and biobanking protocols will similarly optimise variation in novel biomarker results. In addition, strong international collaborative networks and large accessible biobanks are now available within the society [43], and in other global initiatives, to optimise biomarker discovery studies, facilitating independent validation.

## Gaps in the current biomarker development process

Having addressed these major problems, we gain more insight into other shortcomings of the current biomarker development processes [44], the “hypothesis gap”, the “technology translation gap” and the “interaction gap”.

The **hypothesis gap** is the continuous problem that the cause and initial events of the majority of neurological diseases are still not elucidated. Post-mortem evaluation of affected tissue is a powerful tool to define pathologies and possible molecular mechanisms, and several hypotheses for these have been proposed. By definition, however, the earliest preclinical stages cannot be unravelled as follow-up clinical or even downstream pathological events cannot be monitored. In vitro and animal work and familial vs. sporadic disease studies are necessary to strengthen the hypothesis gap. However, for direct investigation of body fluids in the earliest stages of neurological diseases, the biomarker field has employed “omics” technologies. These allow unbiased analysis of large ranges of DNA, RNA, proteins or metabolites, and thus allow identification of biomarker leads as well as novel hypotheses through multimodal pathway analysis [45–48]. The currently most powerful proteomics technologies are based on mass spectrometry methods. These undergo rapid development, even though the current shotgun proteomics approaches require a low sample throughput, low sensitivity in CSF (detection ranges are typically higher than nanograms per millilitre, too low to allow detection of the current diagnostic AD biomarkers in CSF) and relatively large within and between experimental variation. The usual practise in protein biomarker development studies is to employ antibody-based assays for clinical validation of novel identified protein biomarkers. Immunoassays are typically more sensitive, can easily be used for high sample throughput and are broadly implemented in routine clinical chemistry. However, specific and sensitive immunoassays are not available for the majority of novel biomarker proteins, especially if they correspond to post-translationally modified isoforms, and development of novel assays is costly and is conceived as high risk. In contrast, targeted mass spectrometry assays are based on the same technological principle, but this technology still needs further optimisation and automation before robust and large scale routine use is within reach. Similarly, RNA markers are likely easier to validate from the technological perspective, yet the methods still need optimisation for application for CSF analysis, while large scale metabolomics using CSF is still in its infancy [14].

This high risk leads to the second gap, **the cross-technology translation gap**. Mass spectrometry has been the method of choice for unbiased analysis of large numbers of proteins in usually small numbers of patients, but for large scale validation of a handful of proteins in large numbers of patients and clinical implementation, immunoassays are the method of choice. This requires a cross-platform translation, which is an important hurdle. Mass spectrometry identifies peptides from experimentally fragmented proteins while immunobased assays detect natively folded proteins and protein isoforms. Thus, validation of proteins identified by one technology by a completely different technology means, by definition, that a large gap needs to be bridged. This is even more relevant for pathological conditions, where proteins can have lost their original configuration (e.g. during aggregation) and be proteolytically spliced [49]. One solution to overcome this technology translation gap is to start discovery by immunobased methods, e.g. immunobased mass spectrometry [50], or applying immunobased arrays for protein biomarker discovery, such as antibody-based arrays (e.g. Luminex, proximity ligation assays [51, 52]). This will be a less unbiased approach, yet is usually more sensitive, and allows replication of original findings in smaller panels of selected differentially regulated proteins. Other solutions to bridge both the hypothesis gap and the cross-technology translation gap is more extensive verification of potential novel identified biomarkers by merging of omics datasets obtained by different centres, different technologies and different matrices (CSF-tissue-blood). Alternatively, the analytical conditions applied during analysis in one technology (for example mass-spectrometry or antibody development) may differ from those in the sandwich development, and more insight at every step of this process, as well as epitope exposure in native proteins, will help to bridge the cross-technology validation of novel biomarker leads.

**Fig. 1:**
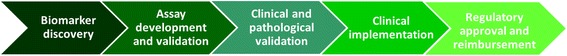
Steps in the biomarker development process

The third identified gap, the **interaction gap**, is defined as the need to constantly discuss and adjust the study design throughout the lengthy and multidisciplinary process of the biomarker development process. We define five essential steps during this development process, involving different disciplines that each speak their own language (Fig. 1). The first step is discovery, with the mentioned omics technology tools or based on a priori hypotheses (the candidate biomarker approach based on pathology and/or previously reported candidate biomarkers). The second step is analytical validation by alternative biomarker assays, which needs to bridge the technology translation gap. When this gap is bridged, which is a high risk undertaking, extensive analytical validation [40] along with proof of concept of clinical use in small cohorts is needed to verify the omics findings. The third step is clinical validation, which typically includes validation in independent cohorts, independent centres and various clinical groups that are relevant for differential diagnosis. The fourth step is clinical implementation, which involves development of in vitro diagnostic tests, establishment of reference material, reference methods and quality control programs, incorporation in clinical guidelines and education of physicians and laboratory practise. Other aspects involved in implementation include evaluation of the role of the biomarker in the diagnostic process, the method of cut-off calculation and communication of the results. The last step includes obtaining regulatory approval and reimbursement by insurance companies.

In all phases, defining the needs of the next steps of the lengthy development process is of extreme relevance. For example, assay performance parameters will change (i.e. become more stringent) during the different developmental stages. In addition, the medical needs at the start of the process could have changed by the time of clinical validation, due to the emergence of alternative diagnostic tools. Public or patient perceptions of the method to collect the fluid can change, very relevant for CSF, which was sometimes perceived as invasive and associated with a high risk of severe complications. Nowadays, it can be well addressed by improving the procedure, avoiding risk factors and providing better information [53]. Other influences leading to a change in the need of biomarkers are breakthroughs in drug development, which can e.g. require more specific or more sensitive biomarkers or require novel targets. In clinical implementation, other aspects of biomarker analysis become relevant, such as the cost of the analysis, turnaround time, readiness of the field to modify clinical guidelines and clinical practice.

## Benefits of filling the gaps

Since the optimal performance of all five steps of the development process are essential and can define its success, it is of utmost importance that the process is approached in its entirety, like a coherent pipeline. Therefore, intensive interaction between all parties involved is required. This is currently not the case, as there is no platform where all the players naturally convene or collaborate. For example, discovery is traditionally the realm of academia and biochemists, as academia has access to cohorts and is in the position to take the high risk involved. Initial assay development for novel biomarkers usually occurs in academia, again due to the high risk. The next step in development, i.e. increasing production and compliance with high quality standards for clinical validation and implementation is the realm of diagnostic companies. Yet, for this validation collaboration with the academic partners is still needed for access to relevant cohorts stored in clinical biobanks. Lastly, implementation in guidelines needs the advocacy of lead physicians, but also requires the involvement of communication specialists. Thus, it is our conviction that biomarker development can be accelerated by continuous and open interaction, to define and adjust the optimal study designs and technical requirements of the biomarkers.

The lack of cross-talk and limited awareness of the essential steps in biomarker development leads to situations where -omics findings can be introduced in the highest impact journals, claiming routine implementation. In fact, the selection of the patient groups, lack of clinical validation and use of low throughput processing technologies warrants greater caution before making such far-fetched claims.

## Novel solutions: the Society for CSF Analysis and Clinical Neurochemistry

These issues are high on the agenda of the novel society for CSF Analysis and Clinical Neurochemistry (“CSF society”). This society originated from two large EU-funded projects: a) the joint program of neurodegenerative diseases (http://www.neurodegenerationresearch.eu/) project BIOMARKAPD (http://www.neurodegenerationresearch.eu/initiatives/annual-calls-for-proposals/closed-calls/biomarkers-transnational-call/results-of-biomarker-call/biomarkapd/), aimed at optimising all aspects of biomarker analysis of biomarkers for Alzheimer’s disease and Parkinson’s disease, and b) the SOPHIA project http://www.neurodegenerationresearch.eu/?s=sophia), with almost similar goals but with a focus on motor neuron diseases. The participants of these projects saw an immediate need for further development of the medical disciplines of CSF biomarker development and clinical neurochemistry beyond a specific call. In addition, the Biomarkers in MS network, which has existed since 2008, joined the initiative.

The main aims of the CSF society are therefore to exchange high level international scientific experience, to facilitate the incorporation of CSF diagnostics into clinical practice and to give advice on inclusion of CSF analysis into clinical guidelines.

The society interacts intensively with stakeholders, such as related industries (e.g. biomarker discovery technology providers, (diagnostic) assay industry), with the perspective that the availability and continuous improvement of high quality technologies and assays is an important need for biomarker development and clinical implementation. Moreover, it stimulates interaction with regulatory bodies, such as IFCC or EMA, and patient organisations, such as ISTAART or Alzheimer Europe, to be able to address patient perspectives relevant during biomarker development (which medical need to address?) and implementation (what information do patients wish to have before undergoing a lumbar puncture?). Stakeholders are invited to participate in the symposia and teaching programs (e.g. giving lectures, discussion panels).

The disease scope of the society is broad, addressing body fluid biomarker research in, for example, dementias, ALS, Parkinson’s disease, neuroinfectious diseases, neoplastic and paraneoplastic diseases, stroke, Creutzfeldt-Jakob’s disease, and autoimmune diseases such as multiple sclerosis, illustrated by the program of its first symposium in 2016. This broad scope is natural in view of the completely identical technical aspects of the biomarker process and its current inherent problems, and many shared biomarkers, for example neurofilaments and inflammatory markers.

As may be clear from the above, the society is a network organisation with a broad disease scope and a specific subject (body fluid biomarkers), which differentiates it from research or cohort consortia with a single-disease scope (e.g. ADNI) or funding organisations (e.g. MJ Fox Foundation, ISTAART).

The current primary matrix of interest of the CSF society is CSF, due to its close contact to the brain tissue and because it has been shown to be informative and useful for specific diagnosis of neurological diseases. The secondary matrix of interest is blood, which is the matrix of choice for monitoring disease severity and progression and treatment efficacy. However, other matrices also are also of interest to the society.

## Strategies of the CSF society

The society employs several strategies to create a new generation of scientists, namely scientists who are able to cross disciplinary borders and take novel avenues and unconventional approaches, to highlight and apply the necessary improvements to optimise and accelerate biomarker development.

The primary activities of the upcoming 5 years are to establish a high quality educational program, including teaching courses, and an educational program for a person to become a certified CSF expert. Another immediate main 5-year target is to organise series of symposia bringing together experts covering the variety of neurological diseases, which functions as a forum for discussion and guideline development for biomarker-related issues (such as guidelines for pre-analytical processing of CSF). Another goal is to develop roadmaps for biomarker development, in which all stakeholders closely interact, to accelerate the biomarker development process and make effective use of available resources in terms of patient biosamples within the society, reagents, expertise and funding. In this way, the society aims to bring in expertise covering the whole development chain outlined in Fig. 1, as it is our vision that this is essential to bridge the current and future identified gaps and to optimise biomarker development.

The educational program of the society contains introductory courses on basic CSF analysis, the state of the art of biomarkers in different diseases, but also hands-on courses to practise interpretation of pathological cells in CSF or to practise novel state of the art ultrasensitive immunoassays.

The advanced courses are supervised by dedicated mentors and include internships and exchange programs for diagnostic analysis and interpretation with the aim to build up scientific and clinical portfolios for the current and next generations of experts on fluid biomarkers for neurologic diseases.

The society is funded by membership contributions. The symposia are supported by sponsorship from industrial stakeholders.

## Conclusions

The goal of the CSF society is to optimise biomarker development by bringing together the necessary expertise and finding the best possible strategy to accelerate biomarker development. We expect to be able to bypass the “valley of death” in which initial promising findings often end, enabling them to bridge the identified hypothesis gap, technology translation gap and interaction gap. We hope that we will thus generate body fluid biomarkers in the next decade for every unmet clinical need, to increase our insight into the biology of neurological diseases and deliver clinically useful tools.
